# Sex differences in early communication development: behavioral and neurobiological indicators of more vulnerable communication system development in boys

**DOI:** 10.3325/cmj.2019.60.141

**Published:** 2019-04

**Authors:** Shir Adani, Maja Cepanec

**Affiliations:** 1 Medical Studies in English, University of Zagreb School of Medicine, Zagreb, Croatia; 2 Child Communication Research Laboratory, Department of Speech and Language Pathology, Faculty of Education and Rehabilitation Sciences, University of Zagreb, Zagreb, Croatia

## Abstract

Perhaps due to different roles they have had in social groups during evolution, men and women differ in their verbal abilities. These differences are also (if not even more) present in children, both in the course of typical and pathological development. Beside the fact that girls have a well-documented advantage in early language development, almost all developmental disorders primarily affecting communication, speech, and language skills are more frequent in boys. The sex-related difference in the prevalence of these disorders is especially pronounced in autism spectrum disorder (1 girl for each 4-5 boys is affected). The aim of this review is to present the sex differences in typical communication and language development and in the prevalence of communication-related neurodevelopmental disorders. Also, a special focus is put on data from the field of neuroscience that might provide insight into the neurobiological mechanisms that can add to the understanding of this phenomenon. We argue that the functional organization of the female brain gives women an inherent advantage in the acquisition of communication and language system over men.

Among all species on Earth, humans have a unique capability of communication using a symbolic communication system, ie, verbal and written language. This ability has helped humankind to thrive and gave it numerous advantages over other species. The highly sophisticated language enabled humans to communicate in a very precise and complex manner, allowing an effective organization within the tribe. Human language complexity can be illustrated by the fact that an average 20-year-old has a vocabulary of approximately 42 000 words (1).

Such a complex communication system is the backbone of our widely variable social relationships. The “social brain hypothesis” stresses the importance of social connections for the overall phylogenetic brain development, as well as for the development of many complex cognitive abilities, which allow us to use symbolic communication systems. According to this hypothesis, the “cognitive demands of living in complexly bonded social groups” are the driving force that led the human brain to increase in size and develop complex functions (2). Moreover, the hierarchy within the social groups led the group members to assume different social roles. Generally, female primates were typically engaged in child raising, food gathering, and domestic tool construction, whereas men tended to hunt and kill. It is believed that “women’s work” contributed to the functional evolution of speech areas, while “men’s work” contributed to male visual-spatial superiority (3).

Many studies confirmed superior verbal performance in women (4,5). Differences between men and women were confirmed not only for the first language acquisition, but also for the acquisition of a foreign language (6). Studies in other primates suggest that sex differences in human communication and language skills might result from the differences between males and females in social tendencies that are “fundamentally rooted in our biological and evolutionary heritage” (7). For instance, sex differences in social tendencies are documented in wild infant chimpanzees (7). Similarly, female newborn macaques look at conspecifics’ faces more than males, and at 4–5 weeks exhibit more affiliative behaviors including gesturing, looking, and proximity to familiar and unfamiliar human caretakers (8). Free-ranging adult rhesus macaques showed sex-related differences in the production rate of social vocalizations, confirming that females rely more on vocal communication, which may have important implication for language evolution theories (9).

However, it is important to notice that differences in human verbal performance are quite subtle and not systematically found on different verbal tasks, with variable results in different age groups (10).

## Sex differences in typical communication, language, and speech development

It has long been known that boys and girls differ in the rates of language development. More than 60 years ago, McCarthy (11) noticed that “these differences are seldom statistically significant, but the careful observer cannot ignore the amazing consistency with which these small differences appear in one investigation after another, each being conducted by a different experimenter, employing different techniques, different subjects, and sampling different geographical populations.“ A recent systematic literature review (12) confirmed the existence of sex differences, but also pointed that they are limited, and often interact with a variety of factors, such as age and task. Generally, differences decrease with age (13), although some studies show the contrary and emphasize that the effect size of sex on toddlers’/children’s/adolescents’ language largely depends on their age and the language aspect (14). Importantly, all significant effects were in favor of girls.

It seems that boys have “weaker” or “slower” capacities for language acquisition. Boys represent more than 70% of late talkers and just 30% of early talkers (15). Studies on early language development (first three years of life) find systematic differences between boys and girls in the process of early communication development and language acquisition. These differences are not observed only in the development of language system, but also in the development of overall social communication skills. Boys lag behind girls in the development of many communication features – eye contact (16), gesture use (17), gesture imitation (18), joint attention (19), social referencing (20)_,_ etc. However, although differences in language development might be a by-product of differences in the development of overall social communication system, sex differences in early language and speech abilities have been most broadly studied.

During the first years of life, girls on average acquire language faster than boys and have larger vocabulary. For example, at 16 months, girls have a vocabulary of 95 words, while boys have a vocabulary of 25 words (21,22). A similar pattern is confirmed in the acquisition of various languages (23,24), in both comprehension and production, as well as in lexical and grammatical development (15). For example, boys produce word combinations on average 3 months later than girls (17). The greatest differences between sexes are noticed at the points of development when children master new communication and language skills.

## Sex differences in the prevalence of communication, language, and speech pathology

Pathologies of communication, speech, and language are diverse in their etiology, prognosis, and presentation. In clinical settings, it is important to differentiate between disorders that primarily affect communication, those that affect language, and those that affect speech. Although these terms sound similar and interchangeable, communication skills (the knowledge of how to exchange messages, both verbally and non-verbally) are fundamental for the acquisition of language (system of symbolic signs used for exchanging complex messages). Speech, as the last “link” in developmental chain, represents the motor production of the language system (production of acoustic signal) and is just one of the possible ways of expressing language (other include writing, signing etc).

One of the most intriguing phenomena in the setting of communication, language, and speech impairments is that male sex is a strong risk factor for the mentioned pathologies, while female sex is a protective factor. This finding is highly replicable and has been observed in countless studies and epidemiological reports (Table 1). Each disorder under the criteria of communication, language, and speech is (a few times) more prevalent in men than in women. The results are consistent over many decades, and across many regions and populations around the world.

**Table 1:** Epidemiological data on sex differences in the prevalence rate of various conditions and disorders affecting communication, language, and speech development in children

Developmental domain	Condition/disorder	Prevalence in general population (%)	Increase in prevalence in boys	Age (years)	Sample size (N)	Reference
Communication, language, and speech	Children with special speech, language, and communication needs (during schooling)	1.6	**2.6**	5-16	6 170 000	Lindsay and Strand (25)
Communication	Autism spectrum disorder	1.7	**4.0**	8	325 483	US Center for Disease Control and Prevention (26)
		2.5	**3.6**	3-17	43 021	Kogan et al (27)
		0.9	**6.3**	7-9	10 138	Narzisi et al (28)
	Social (pragmatic) communication disorder (earlier: pragmatic language disorder)	7.5	**2.6**	4-5	1396	Ketelaars et al (29)
Language	Late language emergence	13.4	**2.4**	2	1766	Zubrick et al (30)
	Language delay	9.6	**2.0**	2	8386	Dale et al (31)
	(Specific) language impairment/disorder	7.4	**1.3**	5-6	7218	Tomblin et al (32)
		7.6	**1.2**	4-5	7267	Norbury et al (33)
Reading	Reading impairment/disability	5.0	**1.6-2.4**	7-8	491 103	Quinn and Wagner (34)
		7.7	**1.4-2.3**	8-10	1 133 988	Wheldall and Limbrick (35)
	Dyslexia	15.6	**1.5**	7.5-12.5	1619	Jiménez et al (36)
Speech	Stuttering	2.5	**2.7**	2-5	3164	Proctor et al (37)
		5.2	**1.7**	2-11	1042	Mansson (38)
	Speech delay	3.80	**1.5**	4-6	1328	Shriberg, Tomblin and McSweeny (39)
	Speech/sound disorders	1.1	**2.1**	6-12	1619	McKinnon et al (40)

The most striking difference between girls and boys is the prevalence of autism spectrum disorder (ASD). Boys/girls ratio in most studies is between 4:1 and 5:1 (26,27), with some studies reporting an even higher ratio (6.3:1) (28). According to “the extreme male brain theory of autism” (41), ASD is the extreme case of the differences in the way male and female brain process social stimuli. Some new behavioral studies in children also highlighted that “sex differences in young children with ASD do not appear to be ASD-specific but instead reflect typically occurring sex differences seen in children without ASD” (42).

These data add additional weight to the hypothesis mentioned earlier – that functional connectivity of the neuronal networks of male brains tends to have diminished capacity for social stimuli processing, and that men are therefore more prone to communication disorders.

Differences between boys and girls in the language domain are also present, but are substantially less pronounced. Boys have a greater risk for late language emergence (2.4:1) (30), but specific language impairment (a disorder when only language system is affected, with typical development of cognitive and communication skills) is just slightly more prevalent in preschool boys than in girls (20%-30% higher) (32,33). Similarly, dyslexia has a prevalence ratio of 1.5:1 in the population of school children (36). Although these discrepancies are significant and should not be understated, they are considerably smaller than differences in the prevalence of communication disorders (ASD and social communication disorder).

Differences in speech pathology are quite variable, but also harder to interpret, because speech production is a motor activity, deeply dependent on fine motor skills, which also differ between sexes in the preschool period (43). Greater prevalence of speech disorders in boys is documented not only when it comes to “typical” articulation problems, but also when it comes to stuttering (37,38) and childhood apraxia of speech (44).

Overall, although documented sex differences in the prevalence of various communication, language, and speech-related conditions and disorders vary in different studies (sometimes largely), data consistently show (almost without any exceptions!) that boys have a significantly higher prevalence of all the conditions affecting communication, speech, and language.

## The role of sex hormones in the communication, language, and speech development

Neuroscientists have been trying to describe the neurobiological foundations of behaviorally documented sex differences in the communication, speech, and language skills. Most studies focused on the role of sex hormones, since they are one of the most “logical” biological markers.

Experimental studies in animals have shown that sex hormones produced in fetal and neonatal life lead to sex differences in neural structure and function. These findings were confirmed in humans, showing that fetal hormones act as an organizing mechanism in the development of regional sexual dimorphism in the brain (45).

Various studies tried to establish the connection between testosterone levels in amniotic fluid and the anatomy of language-related brain areas, as well as functional communication and language skills. Multiple studies reported a strong link between fetal or early postnatal sex hormones levels and communication and language development (16,45-53). Generally, estrogen was found to be correlated with enhanced social and verbal skills and to promote the growth of language centers and related areas in the brain, while testosterone had the opposite effect. Cambridge Child Development Study revealed that fetal testosterone was inversely associated with social development, language development, and empathy in children (16,46) and that elevated fetal testosterone levels were positively associated with autistic traits (47).

Friederici et al (48) showed the effect of testosterone on language organization in 4-week-olds – girls and boys with low testosterone levels showed phonological discrimination effect, while boys with high testosterone levels did not. Also, another study observed negative correlations between testosterone concentrations and babbling at 5 months (49).

A study targeting children aged 8-11 years found a correlation between increased levels of testosterone in amniotic fluid and reduced gray matter volume in the left superior temporal gyrus (Wernicke’s area) and several additional language-related areas, such as a part of Broca’s area (45). Along with the anatomical changes induced by testosterone, increased prenatal exposure to testosterone in boys was also correlated with smaller vocabulary by the age of 2 (50). Testosterone was also suggested to be involved in sex-related hemispheric lateralization (51). Unlike testosterone, higher levels of estrogen measured in 5-month-old children were correlated with better language performance both in boys and girls at the age of 4-5 (52).

Therefore, prenatal and neonatal testosterone exposures are strong candidates for having a causal role in sexual dimorphism in human behavior, including social development, and as risk factors for conditions characterized by social impairments (53).

When considering the adult population, a unique group that contributed to the understanding of hormonal effect on language is the transsexual community. A part of the sex conversion treatment is administration of very high doses of opposite sex hormones, thus providing an excellent opportunity to study the influence of sex hormones on the adult brain. Alterations in the sex hormones levels concurrently changed the volumes of language-related brain areas. Female-to-male transsexuals who were receiving high doses of testosterone for 4 weeks experienced a decrease in the volume of gray matter in Broca’s and Wernicke’s areas but, as what seems to be a compensation, had stronger connectivity between Broca’s and Wernicke’s areas (54). A functional study using functional magnetic resonance imaging (fMRI) in transsexuals also found that total language activity was correlated with post-treatment estradiol levels (55).

## Other important findings that might add to the understanding of sex differences in children

Sexual dimorphism indicators have been found in various studies examining brain anatomy, functional brain activation patterns, gene expression, etc. A detailed analysis of all findings from the field of neuroscience is beyond the scope of this article, but here we highlight the most important findings on sex differences in language skills.

Neurogenetic markers of language skills have first been studied in *FOXP2* gene. *FOXP2* seems to enable the unique verbal communication in humans and is one of the most studied genes in language research (56). *FOX2* gene exists in other mammals as well, but humans have a specific variation of the gene. However, even in rat pups, sex differences in ultrasonic vocalizations were eliminated by decreasing the amount of *Foxp2* in their brain (57), which implies that this gene might be a component of the neurobiological basis for sex differences in mammal vocal communication. *FOXP2* in humans, compared to other mammals, experienced two amino acids changes, and it is known that for the development of normal language skills, the human genome is required to have two functional copies of human-specific *FOXP2* (58). Otherwise, certain *FOXP2* mutations induce severe articulation difficulties and linguistic and grammatical impairment (59). Interestingly, in a small sample of 4-year-old children, the amount of FOXP2 protein in the left hemisphere was significantly lower in boys than in girls (57). Other genes that are known to be involved in language development often have a crucial role in modulating certain brain processes, including neuronal migration, cell adhesion, or axon guidance (*ROBO1, ROBO2, KIAA0319, DYX1C1, CNTNAP2*), as well as calcium homeostasis (*ATP2C2*) (60). Further studies are needed to investigate the possible sex differences in the expression of these (and other) genes, but some studies on gene expression in autism imply possible sexually dimorphic pathways (61).

Sexual dimorphism in brain anatomy was described in numerous studies. Regardless of whether the studies focused on “social brain” or “language brain,” sex differences were often (but not systematically!) noticed. For example, multiple studies showed differences between boys and girls in gray matter volume in left Broca’s and Wernicke’s areas. However, it is important to note that these differences were not systematically associated with differences in verbal IQ (62,63). This is an important finding (and there are lot of similar findings in various studies), since it highlights the drawbacks of using volumetric studies as a method of examining functional differences. As Etchell et al (12) stated, it is possible that “sex differences in language task performance, and patterns of brain activity associated with language processing may exist in the absence of structural differences and *vice versa*.” Histological studies are, unfortunately, rare, but one such study showed the existence of sex-specific histological characteristics in the area of Wernicke – dendritic arborizations were slightly longer and the dendritic pattern was more variable in women than in men (64).

Although there are many functional studies on sex differences in the activation patterns of various brain areas during the execution of language tasks in adults (65,66), developmental studies are less common. However, available data do point toward differences in the activation pattern between boys and girls (67-71). For example, fMRI scans revealed significant differences between boys and girls in the activation pattern in language-related tasks in children aged 9-15 years (67). What is even more important is that the differences appeared when language tasks were presented in both visual and auditive modalities. Girls processed language by using more language networks of the brain altogether, regardless of the sensory input, while boys showed more input-specific brain network activity. Overall, girls showed more bi-lateral language processing (68). When interpreting findings from various functional studies, it is important to consider sex, age, and developmental trends, since there are probably sex-specific paths in functional organization during development (69,70), with disparate neuroanatomical trajectories in boys and girls (71).

## Conclusion

Speech, language, and communication skills within typical population are enormously variable. When considering the multiple brain areas and the complexity of cognitive and motor processes involved, the number of years it takes to acquire language or develop communication skills, and the profound and vital role the environment plays in the development of the mentioned skills, it is clear that we are dealing with multifactorial systems that take years to build. However, despite huge interindividual differences that exist regardless of the individual’s sex; men and women, as groups, tend to show systematic differences in communication and verbal abilities. Numerous epidemiological studies (some of which we mentioned in Table 1) found significantly higher prevalence of communication, language, and speech disorders in boys than in girls. Likewise, the normal process of communication and language skills development is faster and more advanced in girls compared with boys.

All the findings presented in this article lead to the conclusion that the neurobiological foundations for developing such complex communication system are more vulnerable and prone to disorders in boys. In this review, we presented a range of studies from the field of neuroscience that offer pieces that might help in solving the puzzle of the mentioned sex differences. Many studies clearly showed the correlation between sex hormones and developmental outcomes. Moreover, anatomical, histological, and brain activation differences in the speech and language brain areas were also documented. Overall, it seems that the functional organization of the female brain gives women an inherent advantage in the acquisition of communication and language system over men. The specific mechanisms that lead and contribute to the development of this advantage are yet to be fully discovered.

**Table:** REFERENCES
